# Preoperative embolization of renal cell carcinoma metastases to the bone prior to stabilization procedures does not result in reduction in intraoperative blood loss

**DOI:** 10.1007/s10585-022-10195-2

**Published:** 2022-11-26

**Authors:** Sebastian Koob, Henrike Schulze-Steinen, Milena M. Plöger, Thomas M. Randau, Anna C. Strauß, Richard Placzek, Andreas C. Strauß

**Affiliations:** 1 Department of Orthopaedics and Trauma Surgery, University of Bonn, Venusberg-Campus 1, 53127 Bonn, Germany; 2 Department of Diagnostic and Interventional Radiology, University of Bonn, Venusberg-Campus 1, 53127 Bonn, Germany

**Keywords:** Transarterial embolization, Skeletal metastases, Renal cell carcinoma, Metastatic disease, Palliative surgery

## Abstract

**Purpose:**

The effect of preoperative embolization of bone metastases prior to stabilization procedures in reducing intraoperative blood loss remains controversial. This study aimed to analyze the effect of preoperative embolization on orthopedic stabilization procedures of the extremities and spine in cases with bone metastases from renal cell carcinomas. In particular, do these patients suffer less blood loss during the operation and do they need lesser fluid replacements or packed red cell bags intra- and perioperatively? Does preoperative embolization reduce the duration of surgery?

**Methods:**

We retrospectively reviewed stabilization procedures of the spine and extremities at our institution between 2011 and 2021 for group differences (embolization vs. no embolization) in terms of blood loss, fluid substitution, need for packed red cell transfusions, tumor size, and duration of surgery.

**Results:**

We reviewed 79 stabilization procedures of the spine (n = 36) and extremities (n = 43), of which 30 included preoperative embolization procedures. Surprisingly, the embolization group showed a statistically significant increase in blood loss, the need for fluid substitution, and red cell transfusions. Subgroup analysis revealed a significant negative effect of preoperative embolization on stabilization procedures of the extremities.

**Conclusion:**

Based on our data, preoperative embolization of renal cell carcinoma metastases of the extremities had a negative effect on intraoperative blood loss and the need for fluid substitution and should therefore be avoided. Our data did not show an effect on stabilization procedures of the spine.

## Introduction

Since the first publication on the preoperative embolization of highly vascularized tumors or bone metastases in 1975 by Feldman et al. [1] the effects and benefits of this intervention remain controversial. In theory, a reduction in blood loss and perioperative morbidity is achieved by obstructing the main supply vessels of these tumors. Many retrospective studies have shown a favorable effect of preoperative embolization on the reduction of intraoperative blood loss and the need for packed red cell transfusions [2–4], while others, including the only randomized prospective trial, showed no statistical relevance of the procedure [5]. However, these studies focused on spinal metastases, while metastases to the extremities have not been examined. The aim of our study was to evaluate the effect of preoperative embolization in patients with renal cell carcinoma, (as an example of a highly vascularized tumor,) treated for skeletal metastases of the extremities and spine. We wanted to answer the following questions: Do these patients suffer less blood loss during the operation (measured in ml), and do they need lesser fluid substitutions or packed red cell bags intra- and perioperatively? And also, does preoperative embolization reduce the duration of surgery in minutes?

## Materials and methods

We retrospectively reviewed 79 surgical procedures in 54 patients who underwent operative stabilization of pathological spinal or extremity fractures due to renal cell carcinoma metastases between 2011 and 2021 at our institution. Patients were assigned to two groups (no preoperative embolization (Group 1) vs. preoperative embolization (Group 2)). Group 1 included 22 spinal metastases (44.9%) and 27 metastases of the extremities (55.1%). Group 2 included 14 spinal metastases (46.7%) and 16 extremity metastases (53.3%). The types of operations performed included spinal decompression with fusion, composite osteosynthesis, and modular endoprostheses. After 2015, preoperative embolization of bone metastases of renal cell carcinoma was introduced as a standard procedure before stabilizing the pathological fractures at our institution. In emergency situations, such as neurological impairment or highly dislocated fractures, no preoperative embolization was performed owing to time constraints. Patient charts and intraoperative documentation were reviewed for demographic information, type of operation, tumor size, embolization figures, surgical duration, intraoperative blood loss, fluid substitution, blood transfusions, and blood loss per minute of operative time. To achieve a more precise value for intraoperative blood loss, the consumption of compresses and abdominal linen was estimated using a mean blood content of 50 and 5 ml for abdominal linen and compresses, respectively. Data were processed using GraphPad Prism version 9.3.1 for Windows (GraphPad Software, San Diego, California USA, www.graphpad.com) for the calculation of descriptive statistics. The Mann–Whitney U test was used for group comparisons. Data is presented in mean ± standard deviation. This study was approved by the Ethics Committee of the Medical Faculty, University of Bonn (Reg-Nr.:241/17).

## Results

**Fig. 1:**
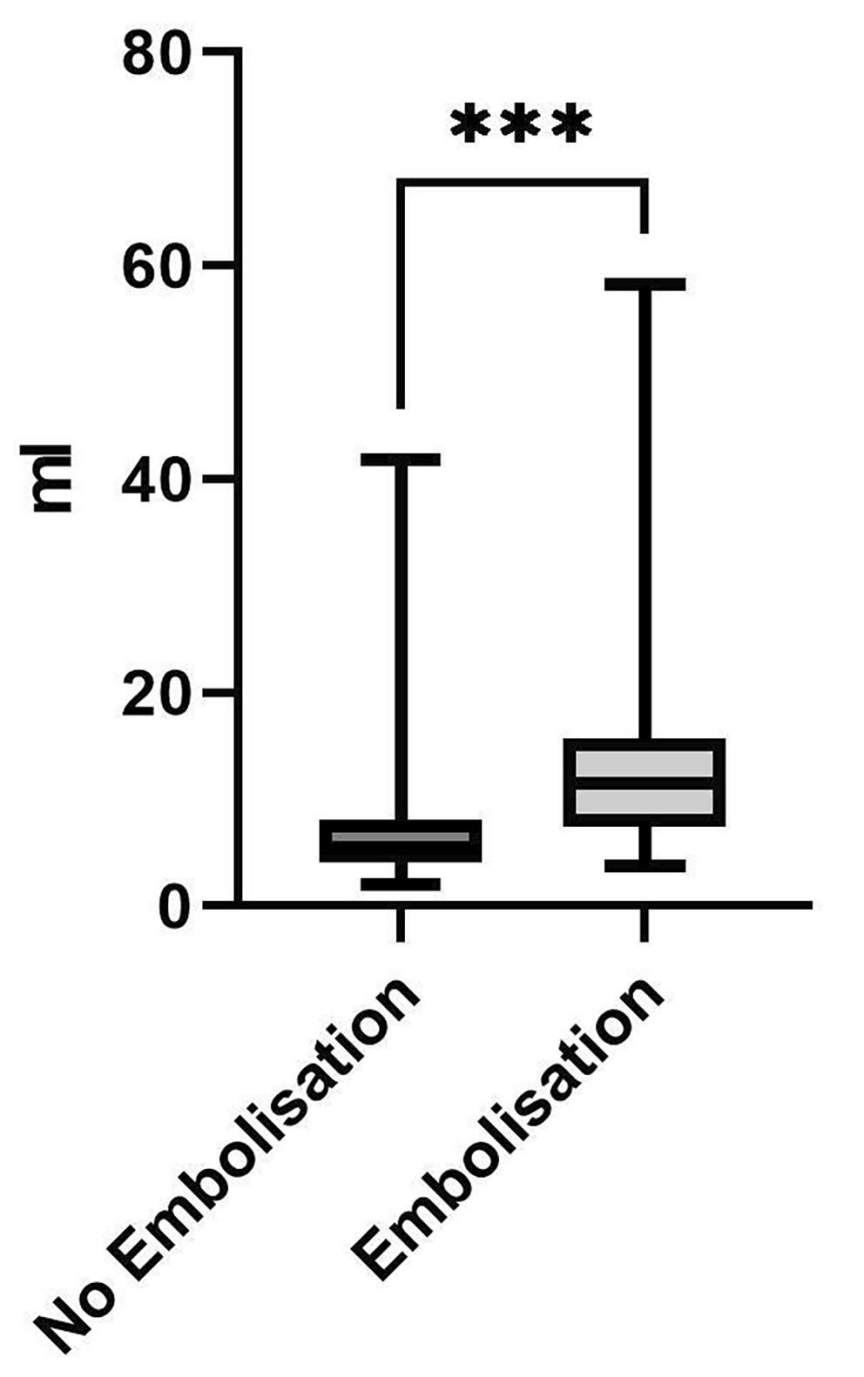
Blood Loss in ml per Minute Operation Time

In this retrospective study, we included 54 patients and 79 surgical interventions for metastases from renal cell carcinoma. In general, there were no important differences between the groups in terms of age (G1 67.82 years / G2 66.20 years, p = 0.5366), gender (G1 24♂/10♀, G2 18♂/9♀, p = 0.7861) and diagnosis. All of the patients were in renal cancer stage 4 according to the American Cancer Society (ACS) with multiple organ and bone metastases. Group 1 (n = 49) received no preoperative embolization; group 2 (n = 30) received preoperative particle embolization of the supply vessels at a mean of 25.6 h (min 2 h, max 92.4 h) prior to surgery. 7 patients received both a preoperative embolization and no preoperative embolization in different surgical interventions., The types of operations performed showed a similar distribution in both groups (Table 1). The volume of blood loss in group 1 (no embolization, suction plus compresses/abdominal linen; 1583 ± 1388 ml) was less than that in group 2 (embolization, suction plus compresses/abdominal linen; 2371 ± 1409 ml; mean difference [MD] -787.4 ± 323.6 ml; 95% confidence interval [CI], 143.0–1432; p = 0.0173). The mean volume of fluid substitution was less in group 1 than in group 2 (3286 ± 2039 vs. 5067 ± 2697 ml; MD -1781 ± 535.2 ml; CI 715.3–2847; p = 0.0013). The mean duration of surgery was not different between groups 1 and 2 (226.3 ± 120.6 vs. 198.6 ± 89.45 min., p = 0.2585). The blood loss per minute operative time was less in group 1 than in group 2 (7.7 ± 6.9 vs. 13.76 ± 11.09 ml/min; MD -6.017 ± 2.023 ml/min, p = 0.0039) **(**Fig. 1**)**. The mean transfusion of 1.4 ± 2.7 packed red cell bags in group 1 was less than that in the 3.0 ± 3.2 bags in group 2 (median difference [Hodges-Lehmann] 3.0; p = 0.0016). The tumor size was retrieved from radiological documents in 15 (50%) cases in the embolization group and in 25 (51%) cases in the no-embolization group. No difference in tumor size was observed between the groups (no embolization vs. embolization, 93.23 ± 85.94 vs. 74.32 ± 71.83 cm³, p = 0.4793).

**Table 1:** Types of Operations performed per Group

Group	Modular Endoprosthesis	%	Composite Osteosynthesis	%	Decompression + Spinal Fusion				Total
					**1 to 2 Segments**	**%**	**3–10 Segments**	**%**	
**No Embolization**	18	37%	9	18%	11	22%	11	22%	49
**Embolization**	11	37%	5	17%	9	30%	5	17%	30

## Subgroup Analysis

Even though the distribution of the different operation types was similar in both groups (view Table 1), a closer analysis of the subgroups to specifically differentiate between spinal and extremity metastases was conducted.

## Spinal metastases

**Fig. 2:**
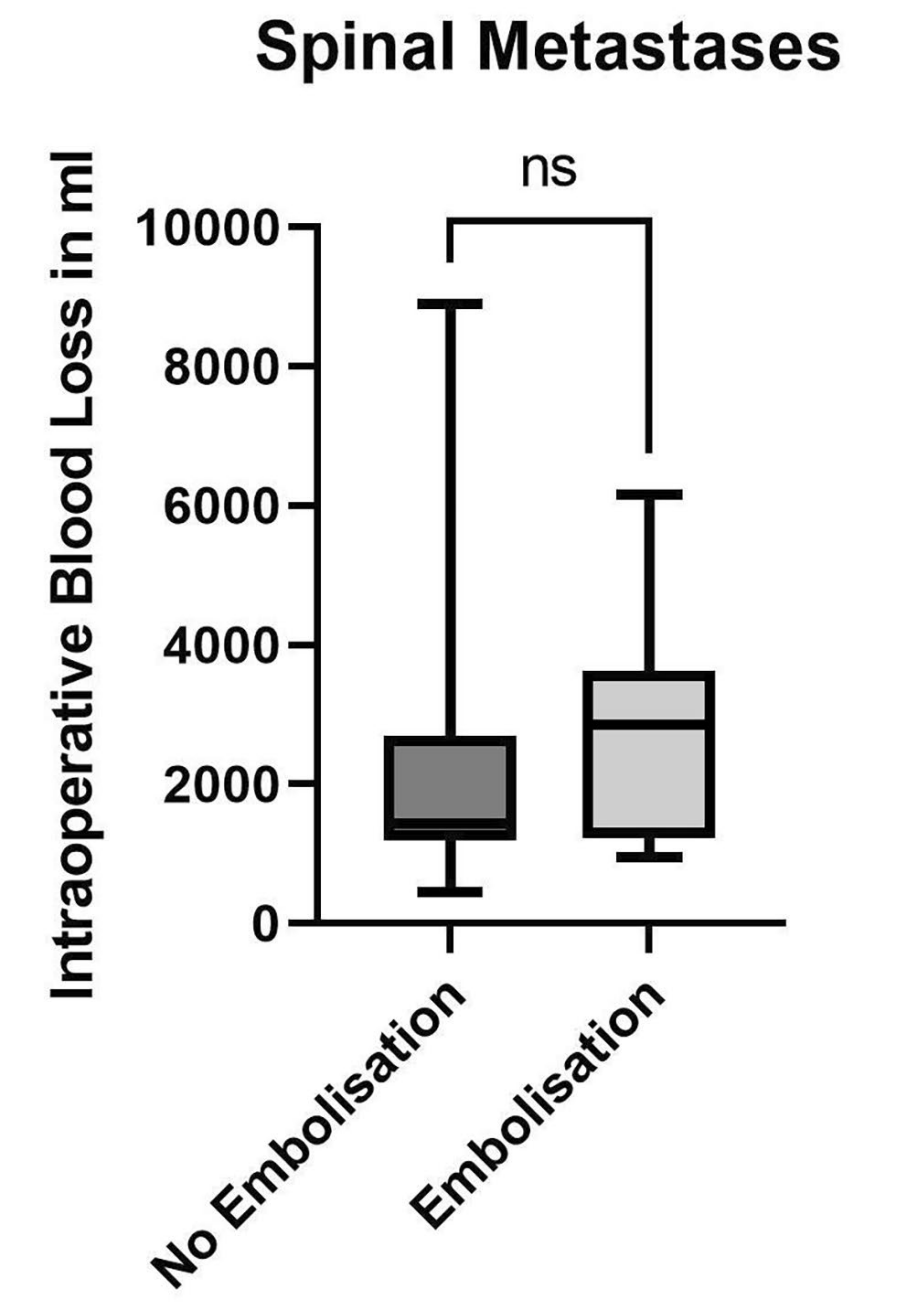
Intraoperative Blood Loss in Decompression + Spinal Fusion Surgery

The mean estimated blood loss within the pre-embolized spinal fusion group (group 2, n = 14; 2770 ± 1523 ml) did not differ significantly from that in the non-embolized spinal fusions in group 1 (n = 22; 2051 ± 1802 ml, p = 0.2251, **(**Fig. 2**)**. The mean fluid substitution in the pre-embolized spinal fusion group within group 2 (5214 ± 2874 ml) did not differ significantly from that in the spinal fusions in the non-embolized group 1 (4227 ± 2313 ml, p = 0.3132). The median operative durations were not different in the pre-embolized and non-embolized groups (178 and 222 min, respectively, p = 0.2115). Overall, preoperative embolization of spinal metastases before spinal fusion did not show any significant effect.

## Extremity metastases

**Fig. 3:**
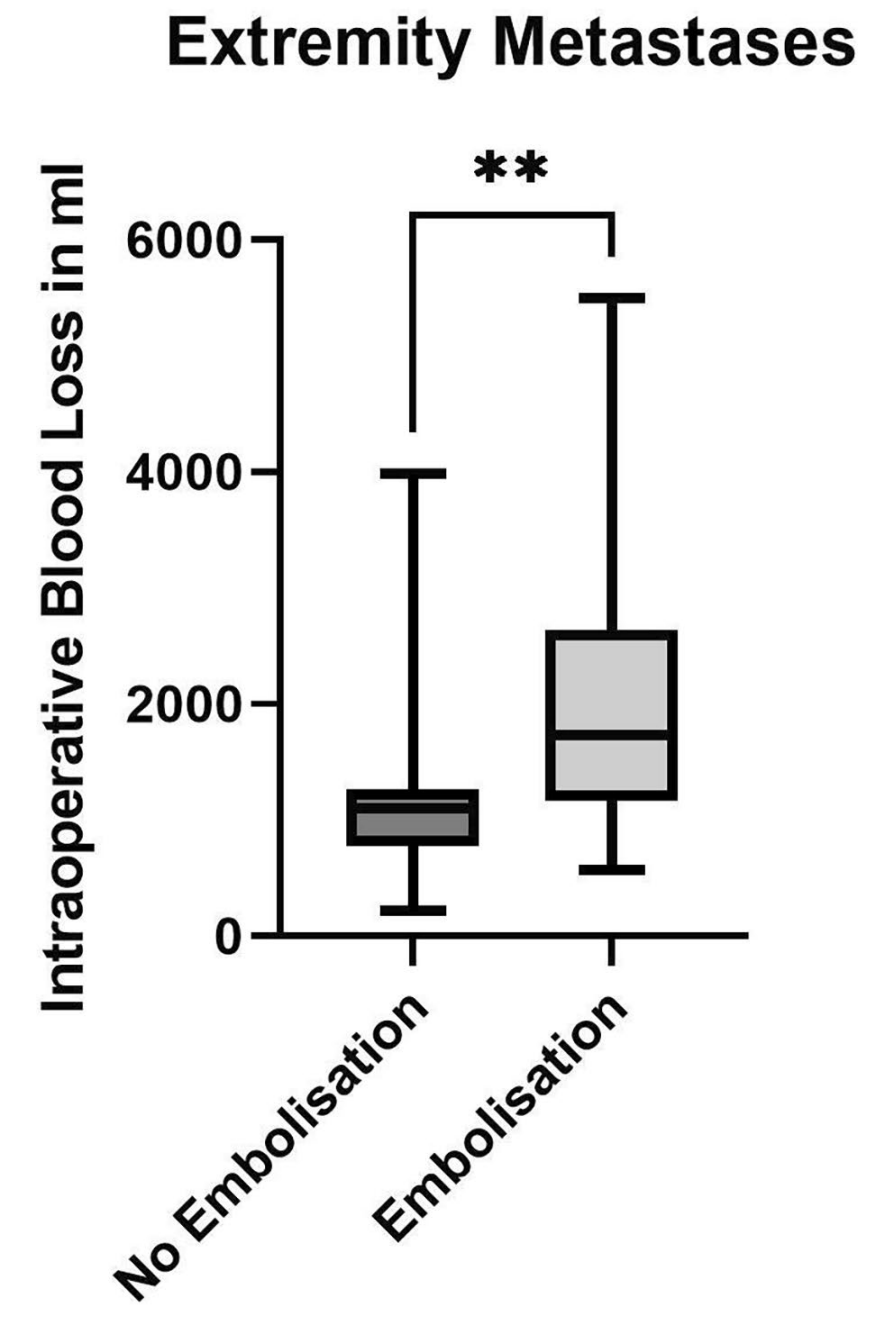
Intraoperative Blood Loss in Stabilization Procedures of the Extremities

The mean estimated blood loss within the group with pre-embolized metastases of the extremities (n = 16; 2022 ± 1244 ml) was higher than that in the non-embolized group 1(n = 27; 1202 ± 774 ml; MD 819.7 ± 306.9 ml, CI 199.9–1440, p = 0.0068; Fig. 3**)**. The mean volume of fluid substitution in the pre-embolized group (4938 ± 2620 ml) was higher than that in the non-embolized group (2519 ± 1411 ml; MD 2419 ± 612.8 ml, CI 1181–3657 ml, p = 0.0012). The median duration of surgery of 184 min in the pre-embolized group (184 min) was not different to that in the non-embolized group (190 min, p = 0.7047). Overall, preoperative embolization of extremity metastases prior to stabilization procedures showed a negative effect on intraoperative blood loss and fluid substitution within an equal operative duration.

## Discussion

Preoperative embolization of highly vascularized tumors and metastases has shown a reduction in intraoperative blood loss and need for packed red cell transfusions in several retrospective studies, while the number of prospective trials is still sparse. In fact, the only available prospective study on this matter showed no favorable effect of preoperative embolization on blood loss and survival. The benefit of the procedure therefore remains controversial. We introduced preoperative embolization of renal cell carcinoma metastases of the spine and extremities in our institution in 2015 and conducted the current study to evaluate the impact of our efforts on blood loss, need for packed red cell transfusions and surgical duration. In our retrospective analysis, we could not detect the benefit of preoperative embolization of renal cell carcinoma metastases in terms of intraoperative blood loss, fluid substitution, operative duration, or need for red cell transfusions. Subgroup analysis revealed a significant advantage of *not* performing preoperative embolization in cases of stabilization of fractured metastases of the extremities, while differences in the blood loss, fluid substitution, and operative duration were not statistically significant in spinal metastasis stabilization.

The most important limitation of our study is based on its retrospective character and also constitutes one possible explanation for our findings. The operating surgeon was aware about whether preoperative embolization had been done or not, and that circumstance may have altered his or her surgical technique as will be explained later. Since tumor size affects the complexity of a surgical procedure, and therefore might increase blood loss and surgical duration, another limitation lies in the fact, that we were able to retrieve the tumor size in only about 50% of the patients under study. However, the initial decision whether to perform a preoperative embolization, was not based on the parameter “tumor size”, but on time resources. The different skill sets of the surgeons is always a decisive and therefore objectivity limiting factor when evaluating surgical performances. From the authors´ point of view, that specific limitation cannot be easily ignored either in retrospective, or prospective studies.

Our first key objective was an answer to the question whether patients who received a preoperative embolization suffered less blood loss and needed fewer red cell transfusions and fluid substitutions. We saw that, while in operative procedures on spinal metastases preoperative embolization did not show any effect on these parameters, in stabilization procedures of the extremities preoperative embolization even had a negative impact. Our findings contradict those of other studies that focused only on the embolization of spinal metastases [6–8] and who favored preoperative embolization. Due to the retrospective design of the study, blinding was not possible. Therefore, one possible explanation for the higher blood loss and necessity of intraoperative packed red cell transfusions and fluid substitution in the embolization group could involve the more invasive and radical surgical technique of the surgeon with knowledge of prior embolization procedures that resulted in a feeling of safety. Another possible explanation could be the bias in favoring larger tumor sizes for preoperative embolization, thus causing higher blood loss due to a more expansive surgery. Although we were unable to retrieve definite tumor size information from all cases (lack of sufficient cross-sectional imaging), we did not observe a significant difference in the tumor size between groups. Rehák et al. also found a higher intraoperative blood loss within the embolized group in a small cohort of 15 patients treated for spinal renal cell carcinoma metastases. However, the study demonstrated a bias towards a larger tumor size and expansive surgery within the embolized group [9]. Theoretically, the vascularity of the metastasis can be a decisive factor in the effectiveness of embolization, but an accurate method for evaluating the vascularity of metastases preoperatively has not yet been developed. The interval between embolization and surgery impacts the reduction of intraoperative blood loss. Most of our patients underwent embolization the day before surgery, while Kato et al. suggested that embolization on the day of surgery had a higher impact [10]. However, these findings do not explain the higher blood loss in the embolization group than in the non-embolization group.

The second objective of the study included the evaluation of duration of surgery Our data did not show a difference between the two groups concerning the length of operation, not even within the subgroup analysis, and looking at extremity stabilization procedures specifically. These finding correspond to Gao et al., who also did not find differences in surgical duration in a meta-analysis of retrospective studies that evaluated effectiveness of preoperative embolization of spinal metastases. Interestingly, in the case of so-called hyper vascular lesions, there seems to be a positive effect not only on limiting blood loss, but also on limiting surgical duration [11]. In our study we did not categorize vascularity of the lesions, partially because there is no consensus on what is considered nonhypervascular and hypervascular.

## Conclusion

Based on our data, preoperative embolization shows no favorable or negative effect on stabilization procedures for spinal metastases of renal cell carcinoma. In cases of metastases of the extremities, preoperative embolization shows a negative effect on intraoperative blood loss and the need for fluid substitution; therefore, it should be avoided. At both metastasis sites, preoperative embolization had no effect on the duration of the surgical procedure. Although the limited number of surgeries did not allow a decisive conclusion against preoperative embolization, this type of intervention should be carefully examined in a prospective, randomized trial setting. Due to the limited number of patients in a reasonable time period at a single center institution, a multi-center study appears more promising.
